# Gatekeeper Training and Minimum Standards of Competency: Essentials for the Suicide Prevention Workforce

**DOI:** 10.1027/0227-5910/a000794

**Published:** 2021-06-30

**Authors:** Jacinta Hawgood, Alan Woodward, Paul Quinnett, Diego De Leo

**Affiliations:** ^1^ Australian Institute for Suicide Research and Prevention, World Health Organization Collaborating Centre for Research and Training in Suicide Prevention, School of Applied Psychology, Griffith University, Brisbane, QLD, Australia; ^2^ Centre for Mental Health, University of Melbourne, VIC, Australia; ^3^ The QPR Institute, Inc., Spokane, WA, USA

**Keywords:** competencies, standards, gatekeeper training, suicide prevention

## Abstract

**Abstract.** Gatekeeper training (GKT) is one of the most widely used suicide prevention strategies. It involves training people who are not necessarily clinicians to be able to identify people experiencing suicidality and refer them to appropriate services. While there is a dearth of research that supports the causal link between GKT and reduced suicide rates, this is likely the result of a variety of factors including training design, definitions of “gatekeepers,” differing populations in which the gatekeeper (GK) operates, and other variables that may influence suicide rates. Despite this, research suggests that GKT improves people's knowledge, skills, and confidence in helping individuals who experience suicidal ideation and enhances positive beliefs about the efficacy of suicide prevention. However, there is no consensus on GK competencies to allow differences in effectiveness between various training programs to be measured, that is, knowledge, skills and abilities, attitudes, and self-efficacy attributes expected of a person resulting from the training. This paper discusses challenges in developing GK competencies. It uses developments in suicide prevention competencies for clinicians, vocational education, and training sector competencies, as well as empirical work in GKT, to propose minimum GK competencies that may be examined for further research and evaluation of programs.

Over 800,000 people die by suicide globally each year (World Health Organization, 2020). In suicide prevention, a key challenge is the identification of suicidal persons who may be reluctant to disclose their thoughts, feelings, or intentions. Gatekeeper training (GKT) is one of the most important components of broader suicide prevention strategies, focusing on training individuals to be competent gatekeepers (GKs; Cross et al., 2010; Isaac et al., 2009). A GK is strategically positioned to recognize a person in crisis, identify behavioral warning signs of suicide, refer a person to help, and perform any other additional capabilities that may help a distressed individual (Burnette et al., 2015; Isaac et al., 2009). Osteen et al. (2014) identified that one of the challenges within GKT is a lack of clarity about who is considered a GK. They proposed a distinction between community GKs, who are people within the community likely to encounter at-risk individuals (e.g., teachers, clergy, and co-workers), and professional GKs, who are health or other professionals who may encounter at-risk people through their worker role. In exploring GK competencies, this paper is focused on the community GK population, while still being informed by GKT developments for professional GKs.

Although GKT plays a significant role in suicide prevention strategies, the evidence surrounding their impact on suicide rates and suicidal behavior is scant and the long-term outcomes of training programs are varied (Holmes et al., 2019; Yonemoto et al., 2019; Zalsman et al., 2016). For example, in their systematic review on long-term efficacy of GKT, Holmes et al. (2019) found that knowledge and self-efficacy training gains are maintained over time, although with some reduction. However, they also found that attitudes toward suicide prevention did not maintain over time and behavioral intention showed a weak training effect. For example, the only study in their review to show sustained outcomes for behavioral intention, at a 2-year follow-up, was by Litteken and Sale (2018), and limitations of these findings are highlighted. These challenges in understanding varied GKT outcomes may be attributed to several factors. First, encompassing a range of suicide prevention initiatives in an overall strategy creates challenges for studying the efficacy of GKTs in isolation. Second, there are differing levels of scientific rigor and evidence-informed content contained in training programs. Third, learning objectives and pedagogical processes in GKT are highly variable across programs. For example, suicide attitudes are complex and may be multifaceted (Aldrich et al., 2014) and how these are addressed in GKT may have a substantial impact on the outcomes of training programs. Last, there are diverse definitions of GKs, and thus, training varies depending on the needs and education of its audience (Osteen et al., 2014).

One way to strengthen research on the effectiveness of GKT is to establish standardized competencies that will enable greater clarity about learning expectations for GKs. Competency as a concept goes beyond the successful learning of standardized knowledge or skills – it embraces demonstrated application of this knowledge or skill in specific roles and situations (Kaslow et al., 2007). Equally important is the theoretical underpinnings that inform GKT intended outcomes. Based on Bandura's (2001) social cognitive theory, Burnette et al. (2015) developed a theoretical model of gatekeeping informed by the existing literature, describing the pathways between training and behavior. The model identifies four factors that may impact intervention-based decision making, which can be enhanced through training. These factors include knowledge about suicide, beliefs and attitudes about suicide prevention, reluctance to intervene (e.g., lack of responsibility and stigma), and self-efficacy. Although individual characteristics (including demographic information and professional background) as well as systemic factors (such as organizational support of GKs' role) have the potential to influence GKT outcomes (Burnette et al., 2015), the four factors in Burnette's model stand well as foundational competencies underpinning the GK role.

Other relevant theoretical work to GKT is the Theory of Planned Behavior (TPB; Ajzen, 1987). TPB asserts that the intention of performing the “intervention” behavior is correlated with attitudes about the intervention, perceived behavioral control, and subjective norms. TPB also provides invaluable guidance on the importance of examining an individual's attitudes toward interventions with suicidal persons, as they relate to future behavioral outcomes which may arise from GKT (Ajzen, 1991). This paper commences with a discussion of suicide prevention competencies for clinicians, vocational education, and training sector competencies, and developments in GKT research to explore potential GK competencies. Based on this, we propose minimum GK competencies that can be examined through robust research design to examine the efficacy of GKT in achieving expected learning outcomes.

## Exploring Gatekeeper Standards of Competency

### Professional Workforce Competencies

In discussing minimum standards for competencies for GKT, existing competencies for the professional workforce are relevant. While it is neither desirable nor feasible for community GKs to develop all the competencies that apply to clinical professionals, there is some overlap. The American Association of Suicidology (AAS) and the Suicide Prevention Resource Centre (SPRC)-led Task Force on Suicide Prevention developed a set of 24 competencies in seven domains of practice applicable to clinicians for assessing and managing suicide risk (Pisani et al., 2011). These domains include attitudes and approach, understanding suicide, collecting accurate assessment information, formulating risk, treatment and services planning, management of care, and legal–regulatory issues (SPRC, 2006). Rudd et al. (2008) identified the implications of these competencies for supervision to help support mastery of skills in a clinical setting.

Other suicide prevention competencies have been proposed for psychology doctoral programs by Cramer et al. (2013). They identified 10 core competencies including self-reflective practice, empathy, risk assessment, focus on immediate intent, determining risk, development of a treatment plan, notifying others, maintaining good documentation, self-care, and understanding the law. La Guardia et al. (2019) extended the application of Cramer et al.'s (2013, 2019) competencies to a wider mental health workforce. Many of these competencies have relevance for community GKs as they address the processes of identification, engagement, immediate response, and referral for help. Community GKs require more of an “identify and refer” focus as opposed to risk assessment, formulation, and treatment responses, given the limits of the role and context they operate in. As such, competencies applicable to both professional and community GKs may include topics on attitudes toward at-risk individuals, the effectiveness of suicide prevention, and confidence and self-efficacy to work with at-risk individuals (Osteen et al., 2014).

### Vocational Competencies

In Australia, vocational competencies have been registered in the national training package for people performing a role in the identification and crisis intervention of people at risk of suicide. This does not exclusively relate to GK roles, that is, roles such as crisis line workers or social service workers are included. These competencies, and associated assessment guides, include the knowledge, skills, and attitudes generally associated with gatekeeping, as well as risk assessment (Department of Education, 2015). Similarly, the US National Suicide Prevention Lifeline has adopted suicide risk assessment and crisis intervention standards (Joiner et al., 2007). These are reflected in a capability framework in the context of crisis center hotline suicide assessment work, focusing on suicidal desire, capability, intent, and protective factors.

### Gatekeeper Programs and Competencies

Identifying and measuring GK competencies is a challenge in the context of what are often brief GKT programs. Some GKT programs are as brief as 60–90 min, and others up to 2 days in length (QPR, n.d.). Still, GKT can make efforts, where practicable, to adopt at least some of the vocational and professional competencies into GKT and promote a “culture of competence.” In addition, exploring relevant training assessment and content can assist in identifying competencies specific to a GK population.

#### Assessment

While research has attempted to examine the outcomes from GKT, few studies have used standardized assessment measures (Miller et al., 2009). This has hampered the ability to compare training program results and identify empirical outcomes (Isaac et al., 2009; Yonemoto et al., 2019). Recognizing these challenges, Albright et al. (2016) developed a standardized assessment tool that assesses the impact of training on both GK behavior and the at-risk person: the Gatekeeper Behavior Scale (GBS). This scale considers that attitudes and beliefs are antecedents to intention, based on three motivational theories of behavior (Ajzen, 1987; Bandura, 1977; Madden et al., 1992). Furthermore, they proposed that the more opportunities and resources a person has, the more perceived control they have. The GBS measures preparedness to act, likelihood to act, self-efficacy, and sense of control, and overlaps well with Burnette et al.'s (2015) model. This study, and ongoing validation work, represents a positive step toward theoretical standardization of measurable GK competencies. Another scale explores a different aspect of GK behavior – an individual's willingness to intervene (Aldrich et al., 2014). The Willingness to Intervene against Suicide (WIS) questionnaire is based on the TPB (Ajzen, 1987) and provides a reliable and valid measure of an important aspect of suicide prevention behavior. This work contributes greatly to the ability to measure important competency outcomes for evaluations for GKT.

#### Training Content

Common objectives of GKT include increasing knowledge, attitudes, and skills in identifying those at risk of suicide, enhancing the ability to identify and respond to a person in crisis, and facilitating help seeking and/or referral (Burnette et al., 2015; Hawgood et al., 2015; Osteen et al., 2014; Quinnett, 2012; Rodgers, 2010). While these core objectives are common across most GKT programs, there remains wide heterogeneity among program content and delivery. If GKT content and learning objectives were aligned with standardized minimum competencies defined specifically for the GK role, better measurement of program outcomes may be possible. To the authors' knowledge, the work by Cigularov et al. (2009) is the first example of an empirical approach to identifying characteristics of a GK. The authors identified the essential competencies as including knowledge of warning signs, knowledge of resources for help and referral, good active listening skills, ability to maintain confidentiality, showing concern about others, and trustworthiness. They also identified that a superior GK would additionally remain calm under pressure and be genuine, sincere, and compassionate.

While GKTs are varied, some programs have identified and trained in specific competencies (QPR, n.d.; Rodgers, 2010). The Question, Persuade, and Refer GKT (QPR, n.d.; Quinnett, 2012) specifically focuses on and assesses attitudes, knowledge, self-efficacy, critical thinking, and judgment, as well as the capacity to engage in expected GK behaviors. Another program, the Applied Suicide Intervention Skills Training, has four intermediate outcomes including: (a) identification of risk, (b) connecting, (c) understanding, and (d) assisting (Rogers et al., 2010). These two programs, while having similar goals, demonstrate the importance of reaching a universally adopted definition of GKT. Minimum competencies in GKT would allow for a comparison across programs.

Further work in Australia has attempted to benchmark minimum competencies for GKs (Hawgood et al., 2006, 2015). This research established suicide prevention training competencies targeted at a broad range of front-line GKs as part of an online suicide prevention training project (Hawgood et al., 2006). The competencies were aligned closely with the AAS competency framework for mental health professionals (Pisani et al., 2011; SPRC, 2006). Furthering this work, Hawgood et al. (2015) identified a set of minimum competencies to examine general GKT outcomes in a subsequent training evaluation project. Their work was based on a combination of current research (Burnette et al., 2015), defined characteristics or competencies of effective GKs (Cigularov et al., 2009), GKT evaluations (Isaac et al., 2009), and Griffith University's existing GKT benchmarks (Hawgood et al., 2006; see the tables in Electronic Supplementary Material 1 for identified learning outcome domains and competencies for suicide prevention workers, respectively).

## Proposed Gatekeeper Competencies

The movement toward defining and measuring suicide prevention competencies for workers in suicide prevention has gained momentum over the past decade (Albright et al., 2016; Aldrich et al., 2018; Cramer et al., 2013; Osteen et al., 2014). However, there remains a need for a well-defined set of minimum set of competencies for GKs universally. This will ensure that robust research into program efficacy at scale and population-based impact can occur. We are proposing that GKs require a core set of minimum competencies reflective of their training and GK role, relating to their suicide prevention knowledge, skills and abilities, attitudes, and self-efficacy.

Using all the existing material, we propose competencies for GKT as outlined in Table 1 that should be explored in future research, as a basis for examining GKT and its effectiveness. We outline knowledge, skills and abilities, attitudes, and self-efficacy that seem to commonly apply across GKT training programs. Application of these competencies as a common minimum addresses the challenge identified in the literature that the skills and abilities involved in GK roles are varied (Yonemoto et al., 2019). This set of competencies will support comparable research on training programs. Variations in specific areas within these competency domains can also be identified, when applicable, for the examination of more tailored training approaches.

**Table 1:** Proposed gatekeeper competencies

Competency	Specific areas of competency
Knowledge	•Knowledge of suicide facts and trends, appropriate/safe language, stigma, and diversity^a^ •Awareness of suicide prevention approaches • Understanding of the complexity of suicidal behavior • Understanding of risk and protective factors • Knowledge of warning signs and their importance for response and intervention • Knowledge of local referral resources • Knowledge of the critical role of lived experience in suicide prevention
Skills and abilities	•Ability to recognize suicidality (including warning signs) •Being able to engage and connect with the suicidal person • Identifying appropriate response(s) to a person in crisis • Strong interpersonal skills • Being able to collaboratively make appropriate referrals • Ability to identify and access resources for help and referral • Ability to maintain confidentiality
Attitudes	•Positive attitudes about the efficacy of suicide prevention (intervening will positively affect the individual) •Positive attitudes toward self-preparedness and likelihood to intervene • Intent to collaboratively intervene • Belief in control over intervention behavior
Self-efficacy	•Confidence in intervention behavior •Ability to identify factors contributing to interventionist negative emotions and well-being • Development of aptitude for personal development and insight • Understanding of the importance of personal management and self-care when working with people with suicidal ideation
*Note*. While the term competency is used in some contexts to refer to assessments of an individual's performance, for this publication the term is used more broadly to refer to the intended outcomes of GKT programs. ^a^Includes understanding of lived experience of suicide, related stigma impacts, appropriate terminology and language use, and the critical place of lived experience in suicide prevention.	

Knowledge has been repeatedly identified as a construct relevant to GKT (Arensman et al., 2016; Cigularov et al., 2009; QPR, n.d.). GKTs have focused on suicide facts and trends, as well as understanding suicidal behavior and the risk and protective factors (Quinnett, 2012; Rudd, 2008). Findings consistently reveal an increase in knowledge about suicide and suicidal behavior as an outcome of GKT (Yonemoto et al., 2019), which may facilitate increased confidence and willingness to engage in intervention behavior (Rallis et al., 2018). Additionally, understanding lived experience of suicide and the critical place it has in suicide prevention reflects critical and emerging GK knowledge relevant for engaging, connecting, and responding to suicidal crisis (Hawgood et al., 2020). Skills and abilities involve different competency facets, including being able to recognize suicidality, being able to engage the suicidal person in an empathic and compassionate manner, and enabling crisis intervention and referral (Gould et al., 2013; QPR, n.d.). These GK abilities have been repeatedly identified as positive GKT outcomes (Cross et al., 2010; Litteken & Sale, 2018; Yonemoto et al., 2019). Positive attitude change following GKT has been identified (Cross et al., 2010; Jacobson et al., 2012), although outcomes on retention of attitude change are varied (Mo et al., 2018; Yonemoto et al., 2019). Considering the research and related assumptions of the GBS (Albright et al., 2016) and the WIS (Aldrich et al., 2014), it is possible that positive attitudes toward suicide prevention are more likely to lead to suicide intervention behavior. Specific dimensions of suicide attitudes have been identified as important for intervening behavior including attitudes about how this behavior will affect the person in suicidal crisis and how intervening affects the interventionist (Aldrich et al., 2014). Finally, self-efficacy refers to an individual's belief in their capacity to execute specific behaviors and reflects confidence in exerting control over motivation, behavior, and the social environment (Bandura, 1977). Enhanced self-efficacy and self-control are likely to positively influence the likelihood of acting (Albright et al., 2016; Burnette et al., 2015). Studies have described an increase in confidence as an outcome following GKT (Arensman et al., 2016; Clark et al., 2010; Rosen et al., 2020), and training improvements should consider self-care as a critical component of self-efficacy. Increasing confidence in one's own emotional and psychological availability to connect and respond to suicidal persons may be achieved via self-care. Cramer et al. (2013) proposed engaging in debriefing and self-care as an important competency for psychologist training in this domain. We propose that GKTs focus on personal development and insight into the importance of self-management (support) when working with those in suicidal crisis to enhance feelings of competence in GKs.

### Future Research and Limitations

This article suggests potential minimum competency standards to be used in research and evaluation, which are required to enable changes in GK intervention behavior and achieve broader impacts on suicide prevention. The current literature on GKT shows some consistency in identifying knowledge, skills and abilities, attitudes, and self-efficacy as potential important training concepts and learning outcome domains. However, without robust research that explores in more depth whether these factors (as standardized minimum competencies) influence suicide prevention, questions remain about their respective contribution to GKT outcomes. For example, more research is required on the assessment of attitude change from GKT, which may be complex due to its multidimensional nature (Ajzen, 1987; Aldrich et al., 2014). Additionally, it is acknowledged that gains in competency may not result in changed behavior. This is a further point of interest for the development of research methods to examine the effectiveness of GKT regarding skills-based delivery mechanisms and translation factors for longer term effectiveness in GKT. Standardized minimum competencies for GKT are also important as a step toward ensuring minimum standards of best practice for GKs, consistent training content, and a quality assurance mechanism for trainers, training authors, and trainees. They will inform the application of robust scientific methodology to investigate the development of community GK skills, knowledge, self-efficacy, and attitudes, and support further research on the impact of GKT on suicide rates.

### Concluding Remarks 

GK competency standards are important for design, delivery, and evaluation of suicide prevention training outcomes. However, the extent to which training impacts can be compared between programs and their outcomes on suicide rates is currently limited. While there are some consistencies across trainings in GK knowledge, skills and abilities, attitudes, and self-efficacy as potential important competencies, there is no standardized or minimum defined set of competencies that might serve as a framework for GKT development and evaluation. We have proposed a starting point for potential competencies that may be examined for further research and evaluation of programs.

## Electronic Supplementary Material

The electronic supplementary material is available with the online version of the article at https://doi.org/10.1027/0227-5910/a000794

**ESM 1.** Table with learning outcome domains for suicide prevention workers and table with competencies for suicide prevention workers in prevention, intervention, and postvention
