# Supplementary material for: Gatekeeper Training and Minimum Standards of Competency: Essentials for the Suicide Prevention Workforce
Source: Crisis. 2021 Jun 30;43(6):516–22. doi: 10.1027/0227-5910/a000794 (PMC9716344; doi:10.1027/0227-5910/a000794)
Supplement: Supplementary file 1 [file cri_43_6_516_esm1.pdf]

https://doi.org/10.1027/0227-5910/a000794

**Electronic Supplementary Material 1**

Table 1. Learning outcome domains for suicide prevention workers

| What the worker is able to 'do': Competence (knowledge, attitudes and skills) | How the worker approaches their suicide prevention practice | The worker as a professional |
|---|---|---|
| • Clinical/applied skills<br>• *Practical procedures/approaches and strategies<br>• *Engaging and assessment of the client<br>• Intervention and management<br>• Health promotion and early intervention (including networking)<br>• *Communication and referral<br>• Handling of information (documentation, reporting) and information retrieval | • With understanding of evidence-based theory and practice, and principles of suicide prevention domains<br>• *With appropriate attitudes, cultural understanding, and ethical and legal responsibilities<br>• With appropriate decision making, problem-solving and clinical/applied reasoning and judgement | • *An understanding of the individual's role in the organisation and within the broader suicide prevention spectrum<br>• *An aptitude for personal development, insight and interpersonal communication, and personal and professional development |

* Represents proposed areas of relevance for 'gatekeepers' undertaking non-clinical training

https://doi.org/10.1027/0227-5910/a000794

Table 2. Competencies for suicide prevention workers in prevention, intervention, and postvention

| *Prevention* | *Intervention* | *Postvention* |
|---|---|---|
| *Research, theory and knowledge*:<br>• *Demonstrate understanding of epidemiological trends, statistics, culture and contexts of suicidal behaviours<br>• *Understand the link between attitudes, stigma, language and knowledge<br>• *Understanding the importance of lived experience in approaching suicide prevention<br>• *Demonstrate knowledge of the multifaceted nature of suicidal behaviour and the differing developmental pathways/trajectories for suicide.<br>• Describe different suicide prevention and response models.<br>• Articulate operational frameworks of practice (individual and organisational)<br>• Identify the differences between targeted and universal suicide prevention strategies.<br>• Define and describe ways to increase effective collaboration and partnerships between community and government services<br>• Identify resources within the community for suicidal persons and their families | *Engaging the suicidal person*:<br>• Differentiate between acute, sub-acute and crisis situations.<br>• *Identify differences in engaging responses for different suicidal contexts (above)<br>• *Discuss appropriate language and interpersonal skills for establishing rapport with suicidal persons<br>• Describe the principles for working with suicidal persons<br>• *Understand the unique factors impacting on suicidal persons<br>• Demonstrate appropriate boundaries between the worker and client<br>• *Understand and demonstrate sensitivity towards suicidal persons from different backgrounds | *Management and rehabilitation*:<br>• Describe essential resources and procedures for long-term management of a suicidal person<br>• Demonstrate understanding of the difference between management and long-term therapeutic approaches |
| | *Risk assessment and duty of care*:<br>• *Describe and report on the range of risk and protective factors and warning signs for suicide across different developmental age groups | *Postvention*:<br>• Understand the principles of postvention (including bereavement and prevention of suicide contagion) |

https://doi.org/10.1027/0227-5910/a000794

| | | |
|---|---|---|
| | • Describe an holistic approach to suicide assessment<br>• Apply different assessment methods for identifying risk state<br>• *Elicit important information from assessment of risk factors to guide subsequent intervention<br>• Define duty of care and workplace obligations.<br>• Demonstrate understanding of legal requirements when working with suicidal people | • Describe the main areas to be considered in the aftermath of a suicide.<br>• Describe any differences between postvention plans applied at the individual, family, and community levels.<br>• Identify the needs of the individual, family and community in a plan, and include the identification of external support agencies.<br>• Describe prevention responses to prevent suicide contagion |
| | *Crisis Intervention*:<br>• Understand the difference between crisis intervention and long-term therapy<br>• Describe the principles of crisis intervention<br>• Assess the appropriate risk state and identify the problem<br>• *Describe and match client needs to services identified with the organisation or referring organisation<br>• Describe intervention strategies different stages of crisis intervention (eg, safety, support, and giving information)<br>• *Demonstrate obtaining commitment from a suicidal person and the issues involved in making a contract with a client<br>• *Outline appropriate referral processes<br>• Demonstrate knowledge of a management plan | *Self Care*:<br>• *Understand the importance of different areas of personal management and self care<br>• Describe principles and practice of supervision<br>• Describe principles and practice of debriefing<br>• Understand the usefulness of worker supervision and debriefing within the organisation<br>• *Describe some of the factors contributing to negative emotions and well-being<br>• *Describe the strategies to improve physical and emotional well-being. |

* Represents proposed areas of relevance for ‘gatekeepers’ undertaking non-clinical training
